# Higher incidence of metabolic syndrome components in vitiligo patients: a prospective cross-sectional study^☆^^☆☆^

**DOI:** 10.1016/j.abd.2019.07.006

**Published:** 2020-02-12

**Authors:** Efsun Tanacan, Nilgun Atakan

**Affiliations:** a Department of Dermatology and Veneorology, Ufuk University School of Medicine, Ankara, Turkey; b Department of Dermatology and Veneorology, Hacettepe University Hospital, Ankara, Turkey

**Keywords:** Metabolic syndrome, Obesity, Vitiligo

## Abstract

**Background/Objectives:**

To investigate the association between vitiligo and metabolic syndrome.

**Methods:**

A prospective cross-sectional study was conducted between 2014 and 2016. Study (*n* = 155) and control groups (*n* = 155) were evaluated for metabolic syndrome according to National Cholesterol Education Program Adult Treatment Panel III and the International Diabetes Federation criteria. Study group was divided into three groups according to their vitiligo area severity index and vitiligo disease activity score values (Group 1: 6.89 for VASI score, Group A: −1–0, Group B: 1–2 and Group C: 3–4 for vitiligo disease activity score respectively). MetS rates according to both criteria were compared between the vitiligo disease activity score and vitiligo area severity index groups.

**Results:**

Metabolic syndrome rates were 37.4% and 40% in the study group and 19.4% and 26.5% in the control group according to National CholesterolEducation Program Adult Treatment Panel III and International Diabetes Federation criteria, respectively (*p* < 001 and *p* = 0.011). Metabolic syndrome was more frequent in vitiligo area severity index Groups 2 and 3 compared to vitiligo area severity index Group 1, and in vitiligo disease activity score Group C compared to vitiligo disease activity score Groups A and B.

**Study limitations:**

Single center experience, absence of more specific oxidative-stress markers and lack of long-term follow-up of the patients.

**Conclusions:**

Frequency of metabolic syndrome was higher in patients with non-segmental vitiligo and the rate was higher in active/severe form of the disease.

## Introduction

Vitiligo is an acquired, progressive disorder of pigmentation characterized by the development of well-defined white macules on the skin.^1^ It is the most frequent cause of depigmentation with an estimated prevalence of 0.1 to 2 percent in both adults and children.^2^ Males and females are equally affected without prominent ethnic, racial or socio-economic differences.^3^ Vitiligo may occur at any age group from early childhood to late adulthood, with a peak incidence in the second and third decades of life.^4^

Although, many theories have been debated for the etiology of vitiligo, the exact cause of vitiligo is still unknown. Genetic predisposition, autoimmunity, biochemical substances, viral infection, melanocyte self-destruction, oxidative stress, neural and melanocytorrhagy are the most common hypotheses in the literature.^5^ The clinical course of vitiligo is variational and lesions may remain stable or progress slowly for years.^1^ Vitiligo Area Severity Index (VASI) and Vitiligo Disease Activity Score (VIDA) may be used for the assessment of disease severity and activation.^6^ Eventhough, there is no cure for the disease, the physicians have some treatment modalities which may halt the progression of lesions and induce varying degrees of repigmentation. Individualized approach based on the patient's age, skin type, the extent, location and degree of disease activity together with the effect of vitiligo on quality of life should be implemented.^7^

Metabolic syndrome (MetS) is an alarming health problem which affects approximately one-quarter of the world's adult population.^8^ Presence of MetS prominently increases the risk of developing type 2 diabetes and cardiovascular diseases which lead to serious complications like myocardial infarction, stroke and death.^9^ On the other hand, the dramatic increase of autoimmune diseases in western countries following the outbreak of obesity is remarkable.^10^ Furthermore, MetS is regarded as a state of chronic low grade inflammation which can result in increased levels of proinflammatory mediators like Tumor Necrosis Factor alpha (TNFα), Interleukin-1 (IL-1), Interleukin-6 (IL-6) Plasminogen Activator Inhibitor-1 (PAI-1), and C-Reactive Protein (CRP).^9^ Thus, the relationship between MetS and autoimmune diseases were investigated in many studies.^10^ There are also publications in the literature which investigated the association between MetS and dermatologic diseases like vitiligo and psoriasis.^11^, ^12^, ^13^, ^14^, ^15^, ^16^, ^17^ Additionally, the role of vitamin B12 metabolism in vitiligo was assessed in some studies.^18^, ^19^, ^20^

The aim of this study is to investigate the association between vitiligo and MetS.

## Methods

### Enrollment of patients and control

We conducted a prospective cross-sectional study at Hacettepe University, Department of Dermatology and Veneorology between October 2014 and March 2016. A total of 310 participants were included in the study (*n* = 155 for the study group and *n* = 155 for the control group). Inclusion criteria for the study group were: (1) Patients with diagnosis of vitiligo (2) ≥18 years of age. Control group was consisted of 155 gender and age matched subjects who admitted to dermatology clinic for complaints other than inflammmatory skin diseases. Exclusion criteria were: (1) Presence of pregnancy or lactation; (2) Patients who have used medications that can effect the metabolic status (like systemic steroid therapy or cyclosporine). Written informed consent was obtained from all the participants, and the study was approved by the institutional ethics committee of Hacettepe University (GO 14/338-03).

### Investigation for metabolic syndrome

Study and control groups were evaluated for MetS criteria. Mean age, gender, frequency of risk factors for metabolic syndrome (presence of diabetes mellitus, Hashimoto’s thyroiditis, hypertension, hyperlipidemia, obesity, rate of smoking and alcohol consumption), laboratory test results [levels of Fasting Plasma Glucose (FPG), High-Density Lipoprotein (HDL), cholesterol, Low-Density Lipoprotein (LDL) cholesterol, total cholesterol, triglycerite (TG), CRP and vitamin B12], vitamin B12 deficiency/replacement rates (the percentage patients with lower levels of vitamin B12 levels than reference ranges and/or taking vitamin B12 replacement during the study period), blood pressure measurements, waist circumference and Body Mass Index (BMI) were compared between the groups. Vitiligo patients were also evaluated for frequency of vitiligo subtypes and mean duration of the disease. Furthermore, mentioned risk factors, laboratory tests results and MetS criteria were compared between the vitiligo subtypes (segmental and non-segmental vitiligo). Venous blood samples were taken from the subjects after 12 h of fasting.

Patients with BMI values ≥30 kg/m^2^ were defined as obese. MetS was defined as the presence of any three of the following five traits: (1) Waist circumference ≥102 cm in men and ≥88 cm in women; (2) Serum TG ≥150 mg/dL or drug treatment for elevated TG; (3) Serum HDL cholesterol <40 mg/dL in men and <50 mg/dL in women or drug treatment for low HDL cholesterol; (4) Blood pressure ≥130/85 mmHg or drug treatment for elevated blood pressure; (5) FPG ≥100 mg/dL or drug treatment for elevated blood glucose, according to the 2004 National Cholesterol Education Program Adult Treatment Panel III (NCEP-ATP III).^21^ However, The International Diabetes Federation (IDF) uses waist circumference as an essential element in definition with different thresholds set for different race/ethnicity groups.^22^ We used both criteria in order to objectively compare our results with the results of various studies in the literature. The diagnosis of vitiligo was made by dermatologists based on the clinical findings. VASI and VIDA scores were used for the assessment of disease severity and activation.^6^ Additionally, the vitiligo patients were divided into 3 groups according to their VASI score values. First we calculated VASI Scores for all 155 study group patients. Then we sorted the VASI score values with increasing order and after that we divided the VASI score values into three subgroups according to number of patients in each subgroup (subgroup 1 = 52, subgroup 2 = 52, subgroup 3 = 51). First subgroup consisted of patients with VASI score values <1.94, second subgroup consisted of patients with VASI score values 1.94–6.89 and third subgroup consisted of patients with VASI score values >6.89. Moreover, the study group were divided into 3 groups according to their VIDA score values (Group A: −1–0, Group B: 1–2 and Group C: 3–4 for VIDA score respectively). Metabolic syndrome rates according to both NCEP-ATP III and IDF criteria were compared between the VIDA and VASI groups. Additionally, VASI and VIDA scores for vitiligo subtypes were compared. Furthermore, mean CRP values were compared between VIDA score groups. Finally, MetS rates were also compared between the study and control groups after excluding the patients with obesity, diabetes mellitus, Hashimoto’s thyroiditis, vitamin B12 deficiency and alcohol consumption.

### Statistical analysis

Statistical analyses were performed with the Statistical Package for the Social Sciences (SPSS.22, IBM SPSS Statistics for Windows, Version 22.0. Armonk, NY: IBM Corp.). The Kolmogorov–Smirnov test was used to evaluate the normal distribution of the data. Normally distributed data were presented as means and standard deviations, while non-parametric data were presented as median (range) values. The independent-samples *t* test and Mann–Whitney *U* test were used to compare the parametric and non-parametric variables between the groups, respectively. Categorical variables were compared using the Chi-square test. The significance level with a *p*-value of <0.05 was determined.

## Results

### Demographic features and clinical characteristics

The demographic features and clinical characteristics of the study and control groups were shown in table 1. There were no statistically significant difference between the groups in terms of gender, mean age, rates of coexisting diabetes mellitus, Hashimoto’s thyroiditis and smoking (*p*-values were 0.811, 0.909, 0.723, 0.197 and 0.814 respectively). On the other hand, rates of hypertension, hyperlipidemia, obesity and alcohol consumption were statistically different between the groups (*p*-values were 0.045, 0.002, 0.025 and 0.013 respectively). Patients with vitiligo had higher rates of hypertension (34.2% vs. 23.9%), hyperlipidemia (57.4% vs. 39.4%), obesity (23.9% vs. 10.3%) and alcohol consumption (32.3% vs. 19.4%) compared to the control group.

**Table 1:** Demographic features and clinical characteristics of the study and control group patients

Variables	Study group (*n* = 155)	Control group (*n* = 155)	*p*-Value
*Gender (n, %)*			0.811
Female	72 (46.5%)	71 (45.8%)	
Male	83 (53.5%)	84 (54.2%)	

*Age (mean* *±* *SD, range) (years)*	37.04 ± 12.07 (18–69)	37.37 ± 12.60 (18–73)	0.909

*Frequency of risk factors for metabolic syndrome, n (%)*			
Diabetes mellitus	5 (3.2%)	3 (1.9%)	0.723
Hashimoto’s thyroiditis	27 (17.4%)	18 (11.6%)	0.197
Hypertension	53 (34.2%)	37 (23.9%)	0.045
Hyperlipidemia	89 (57.4%)	61 (39.4%)	0.002
Obesity	37 (23.9%)	16 (10.3%)	0.025
Smoking	59 (38.1%)	56 (36.1%)	0.814
Alcohol consumption	50 (32.3%)	30 (19.4%)	0.013

### Metabolic syndrome components

Comparison of the laboratory test results, waist circumference, BMI, blood pressure values and MetS rates between the study and the control groups were shown in table 2. There were no statistically significant difference between the groups for mean HDL cholesterol, TG, vitamin B12, waist circumference and BMI values (*p*-values were 0.970, 0.630, 0.953, 0.08 and 0.054 respectively). However, there were statistically significant differences between the groups for mean FPG, LDL cholesterol, CRP, Systolic Blood Pressure (SBP) and Diastolic Blood Pressure (DBP) values (*p*-values were 0.013, 0.031, 0.05, 0.02 and 0.05 respectively). Study group had higher FPG (93 ± 20.82 vs. 87.78 ± 11.40), LDL cholesterol (134.09 ± 31.11 vs. 113.56 ± 24.11), CRP (0.36 ± 0.24 vs. 0.26 ± 0.14), SBP (115 ± 15.50 vs. 109.29 ± 14.67) and DPB (74.83 ± 10.07 vs. 71.38 ± 9.17) values compared to the control group. Furthermore, the rates of vitamin B12 deficiency rate was similar between the groups (41.1% vs. 42.1%, *p* = 0.854). On the other hand rates of vitamin B12 replacement therapy and MetS (according to both NCEP-ATPIII and IDF) were statistically significantly different between the groups. Vitamin B12 replacement therapy rate was 16.1% in the study group and 7.7% in the control group (*p* = 0.036). MetS rate was 37.4% in the study group and 19.4% in the control group according to the NCEP-ATPIII criteria (*p* < 001). Additionally, MetS rate was 40% in the study group and 26.5% in the control group according to IDF criteria (*p* = 0.011).

**Table 2:** Comparison of the laboratory test results, waist circumference, BMI, blood pressure values and metabolic syndrome rates between the study and the control groups

Variables	Study group (*n* = 155)	Control group (*n* = 155)	*p*-Value
*FPG (mg/dL) (mean* *±* *SD)*	93 ± 20.82	87.78 ± 11.40	0.013
*HDL cholesterol (mg/dL) (mean* *±* *SD)*	50.08 ± 10.90	50.13 ± 12.37	0.970
*LDL cholesterol (mg/dL) (mean* *±* *SD)*	134.09 ± 31.11	113.56 ± 24.11	0.031
*TG (mg/dL) (mean* *±* *SD)*	127.00 ± 75.90	123.45 ± 71.85	0.630
*CRP (mg/L) (mean* *±* *SD)*	0.36 ± 0.24	0.26 ± 0.14	0.05
*Vitamin B12 (pg/mL) (mean* *±* *SD)*	245.12 ± 100.86	245.91 ± 130.00	0.953
*Vitamin B12 deficiency rate, n (%)*	62 (41.1%)	64 (42.1%)	0.854
*Vitamin B12 replacement rate, n (%)*	25 (16.1%)	12 (7.7%)	0.036
*Waist circumference (cm) (mean* *±* *SD)*	94.20 ± 13.06	91.72 ± 11.85	0.08
*BMI (kg/m*^*2*^*) (mean* *±* *SD)*	26.28 ± 4.71	25.35 ± 3.71	0.054
*Systolic blood pressure (mmHg) (mean* *±* *SD)*	115 ± 15.50	109.29 ± 14.67	0.02
*Diastolic Blood pressure (mmHg) (mean* *±* *SD)*	74.83 ± 10.07	71.38 ± 9.17	0.05

*Metabolic syndrome rate, n (%)*			
NCEP-ATPIII criteria	58 (37.4%)	30 (19.4%)	<0.001
IDF criteria	62 (40%)	41 (26.5%)	0.011

FPG, Fasting Plasma Glucose; HDL, High-Density Lipoprotein; LDL, Low-Density Lipoprotein; TG, Triglyceride; CRP, C-Reactive Protein; BMI, Body-Mass Index, NCEP-ATPIII, National Cholesterol Education Program Adult Treatment Panel III; IDF, The International Diabetes Federation.

### Vitiligo severity

Non-segmental vitiligo was the most frequent subtype in our study with 144 patients (92.9%). Segmental vitiligo was observed in the remaining 11 patients (7.1%). The mean duration of vitiligo was 121.20 ± 115.04 months. In addition, mean VASI score was 4.054 ± 5.54 (0.093 to 43.3) and median VIDA score was 3 (−1 to 4). Table 3 showed the MetS rates for VASI and VIDA groups according to NCEP-ATPIII and IDF criteria. When we compared the vitiligo patient groups according to VASI score in terms of MetS rates (both for NCEP-ATPIII and IDF criteria), we found statistically significant difference between the groups (*p*-values were 0.006 for NCEP-ATPIII criteria and 0.013 for IDF criteria). MetS was more frequent in Groups 2 and 3 according to both criteria compared to Group 1 (6.45%, 14.8% and 16.1% according to NCEP-ATPIII criteria, 7.7%, 16.1% and 16.1% according to IDF criteria for Groups 1, 2 and 3 respectively). Similarly, we found statistically significant difference between the groups according to VIDA scores (*p* < 0.001 for both criteria). Metabolic syndrome rates was more frequent in Group C compared to Group A and B according to both criteria used (0.6%, 1.9% and 34.8% according to NCEP-ATPIII criteria and 0.6%, 2.6% and 36.8% according to IDF criteria). Furthermore, mean CRP values were statistically significantly different between the VIDA groups (*p* = 0.006). Mean CRP value in Group C was higher than the mean CRP values in Group A and B (mean CRP values were 0.20 ± 0.08, 0.22 ± 0.07 and 0.48 ± 0.26 respectively for the Groups A, B and C). Table 4 showed the rates of MetS between the study and control groups after the exclusion of the patients with obesity, diabetes mellitus, Hashimoto’s thyroiditis, vitamin B12 deficiency and alcohol consumption according to both NCEP-ATPIII and IDF criteria. MetS was still statistically significantly more frequent in the study group according to the both criteria compared to the control group (39.5% vs. 8.7% for NCEP-ATPIII criteria and 40.7% vs. 15.6% for IDF criteria respectively, *p* < 0.001 for both criteria).

**Table 3:** Metabolic syndrome rates for VASI and VIDA groups according to NCEP-ATPIII and IDF criteria

Groups	NCEP-ATPIII metabolic syndrome positive (58/155) (37.4%)	*p*-Value	IDF metabolic syndrome positive (62/155) (40%)	*p*-Value
VASI Groups, *n* (%)		0.006		0.013
Group 1 (<1.94)	10/155 (6.45%)		12/155 (7.7%)	
Group 2 (1.94–6.89)	23/155 (14.8%)		25/155 (16.1)	
Group 3 (>6.89)	25/155 (16.1%)		25/155 (16.1%)	

VIDA Groups, *n* (%)		< 0.001		<0.001
Group A (−1–0)	1/155 (0.6%)		1/155 (0.6%)	
Group B (1–2)	3/155 (1.9%)		4/155 (2.6%)	
Group C (3–4)	54/155 (34.8%)		57/155 (36.8%)	

NCEP-ATPIII, National Cholesterol Education Program Adult Treatment Panel III; IDF, The International Diabetes Federation; VASI, Vitiligo area severity index; VIDA, Vitiligo Disease Activity Score.

**Table 4:** The rates of metabolic syndrome between the study and control groups after the exclusion of the patients with obesity, diabetes mellitus, Hashimoto’s thyroiditis, vitamin B12 deficiency and alcohol consumption according to both NCEP-ATPIII and IDF criteria

Group	NCEP-ATPIII metabolic syndrome positive	IDF metabolic syndrome positive	*p*-Value
Study Group, *n* (%)	34/86 (39.5%)	35/86 (40.7%)	<0.001
Control Group, *n* (%)	10/115 (8.7%)	18/115 (15.6%)	<0.001

NCEP-ATPIII, National Cholesterol Education Program Adult Treatment Panel III; IDF, The International Diabetes Federation.

### Comparison of segmental and non-segmental vitiligo patients

Comparison of demographic features, clinical characteristics, laboratory tests, metabolic syndrome components together with VASI and VIDA scores between the segmental and non-segmental vitiligo patients were shown in table 5. Patients were comparable for most of the parametres. However, significantly higher values were found for CRP, waist circumference, BMI, obesity rate for NCEP-ATPIII criteria, VASI and VIDA scores in the non-segmental vitiligo patients (0.001, <0.001, 0.001, 0.05, <0.001 and <0.001, respectively).

**Table 5:** Comparison of demographic features, clinical characteristics, laboratory tests, metabolic syndrome components together with VASI and VIDA scores between the segmental and non-segmental vitiligo patients

Variables	Non-segmental vitiligo patients (*n* = 144)	Segmental vitiligo patients (*n* = 11)	*p*-Value
*Gender (n, %)*			0.730
Male	78 (54.2%)	5 (45.5%)	
Female	66 (45.8)	6 (54.5%)	

*Age (mean* *±* *SD, range) (years)*	37.06 ± 12.10 (18–69)	37.01 ± 12.20 (18–69)	0.910

*Frequency of risk factors for metabolic syndrome, n (%)*			
Diabetes mellitus	5 (3.4%)	0 (0%)	0.69
Hashimoto’s thyroiditis	26 (18.1%)	1 (9.1%)	0.39
Hypertension	49 (34%)	4 (36.4%)	0.55
Hyperlipidemia	84 (58.3%)	5 (45.5%)	0.30
Obesity	36 (25%)	1 (9.1%)	0.67
Smoking	57 (39.6%)	2 (18.2%)	0.20
Alcohol consumption	45 (31.3%)	5 (45.5%)	0.33
FPG (mg/dL) (mean ± SD)	93.4 ± 1.79	88.18 ± 1.80	0.42
HDL cholesterol (mg/dL) (mean ± SD)	50.01 ± 0.91	50.73 ± 3.5	0.83
LDL cholesterol (mg/dL) (mean ± SD)	134.97 ± 3.32	124.25 ± 10.09	0.35
TG (mg/dL) (mean ± SD)	127.88 ± 6.21	119.09 ± 28.69	0.71
CRP (mg/L)(mean ± SD)	0.37 ± 0.02	0.24 ± 0.02	0.001
Vitamin B12 (pg/mL) (mean ± SD)	244.86 ± 8.67	248.45 ± 23.70	0.91
Vitamin B12 deficiency rate, *n* (%)	53 (36.8%)	4 (36.4%)	0.62
Vitamin B12 replacement rate, *n* (%)	31 (21.5%)	1 (9.1%)	0.46
Waist circumference (cm) (mean ± SD)	95.79 ± 1.02	82.09 ± 2.70	<0.001
BMI (kg/m^2^) (mean ± SD)	26.62 ± 0.39	21.90 ± .66	0.001
Systolic blood pressure (mmHg) (mean ± SD)	115.90 ± 1.2	114.09 ± 5.9	0.71
Diastolic blood pressure (mmHg) (mean ± SD)	75.00 ± 0.83	72.72 ± 3.25	0.47

*Metabolic syndrome rate, n (%)*			
NCEP-ATPIII criteria	57 (39.6%)	1 (9.1%)	0.05
IDF criteria	60 (41.7%)	2 (18.2%)	0.20
VASI Groups, *n* (%)			<0.001
Group 1 (<1.94)	40 (27.8%)	11 (100%)	
Group 2 (1.94–6.89)	51 (35.4%)	0 (0%)	
Group 3 (> 6.89)	53 (36.8%)	0 (0%)	

*VIDA Groups, n (%)*			<0.001
Group A (−1–0)	19 (13.2%)	6 (54.5%)	
Group B (1–2)	35 (24.3%)	3 (27.3%)	
Group C (3–4)	90 (62.5%)	2 (18.2%)	

FPG, Fasting Plasma Glucose; HDL, High-Density Lipoprotein; LDL, Low-Density Lipoprotein; TG, Triglyceride; CRP, C-Reactive Protein; BMI, Body-Mass Index; NCEP-ATPIII, National Cholesterol Education Program Adult Treatment Panel III; IDF, The International Diabetes Federation; NCEP-ATPIII, National Cholesterol Education Program Adult Treatment Panel III; IDF, The International Diabetes Federation; VASI, Vitiligo Area Severity Index; VIDA, Vitiligo Disease Activity Score.

## Discussion

Vitiligo is an acquired pigmentary disorder of unknown origin and it is the most common depigmenting disorder worldwide.^1^, ^2^ It may have a prominent phychosocial impact on the patients and it may lower their quality of life.^23^ However, as the exact cause of the disease has not been revealed yet, no curative treatment is available for now.^7^ Thus, management protocols are mostly conservative and they focus on cosmetic conserns.^7^ On the other hand, recent studies have highlighted a much greater danger for health: the association of vitiligo and the MetS.^11^, ^13^, ^17^ As Pietrzak et al. mentioned in their manuscript, if the existence of metabolic disturbances in vitiligo are proved, the future management of vitiligo patients will change.^11^ MetS increases the risk of developing type 2 diabetes mellitus 5 fold and the risk of developing cardiovascular disease 2 fold over the next 5 to 10 years.^9^ Furthermore, patients with the MetS are at 2 to 4 fold increased risk of stroke, 3 to 4 fold increased risk of myocardial infarction, and 2 fold the risk of dying from these events compared to population without the syndrome regardless of a previous history of cardiovascular events.^9^ Therefore, it is crucial to prevent the serious complications of MetS by lifestyle changes and control of risk factors. Additionally, optimal management of MetS may improve the clinical course of vitiligo.

Alterations in cytokine concentrations, autoimmunity and genetic predisposition are thought to be the main factors behind the pathogenesis of vitiligo.^13^, ^17^ Besides, vitiligo does not only affect the skin but it has many systemic manifestations.^13^, ^17^ Production of autoantibodies in vitiligo may also result in the development of autoimmunological comorbidities, like alopecia areata, autoimmune thyroid disease, Addison's disease, pernicious anemia, type I diabetes mellitus, and myasthenia gravis according to some studies.^5^, ^11^ Moreover, increased levels of proinflammatory cytokines such as TNF-α, IL-1 and IL-6 may lead to insulin resistance and atherosclerosis.^11^ Karadag et al. claimed that insulin resistance and lipid profile changes might occur in patients with vitiligo in their study (decreased levels of HDL cholesterol and increased levels of LDL cholesterol and insulin resistance).^24^ Furthermore, another study by Karadag et al. demonstrated higher levels of homocysteine in vitiligo patients compared to controls.^18^ Homocysteine inhibits tyrosinase, an enzyme participating in melanine synthesis and it is a marker for cardiovascular diseases.^18^ Additionally, Silverberg et al. found that vitiligo patients have higher homocysteine and lower vitamin B12 serum concentrations in their study.^25^ According to recent studies, melanocytes are present not only in the skin and hair follicles, but also in the retinal pigment epithelium cells, in some cells of the inner ear, in some parts of the central nervous system and in the adipose tissue.^11^, ^26^, ^27^ Melanocytes in adipose tissue are thought to take part in anti-inflammatory reactions and in the reduction or binding of reactive oxygen species.^11^, ^26^, ^27^ Randhawa et al. found higher rates of melanogenesis in obese patients.^27^ Hoggard et al. and Hoch et al. found higher levels of a-Melanocyte Stimulating Hormone (a-MSH), that binds to the Melanocortin 1 Receptor (MC1R) on human adipocytes and stimulates melanogenesis in their studies.^28^, ^29^ Thus, Pietrzak et al. concluded that decreased number of melanocytes together with the decreased melanogenesis in the adipose tissue might be the common reason behind the oxidative stress both in vitiligo and MetS.^11^ Page et al. suggested testing agonists of melanin production to prevent development of MetS in their studies.^26^ Likewise, Noel et al. indicates statins as immune-modulating medications for vitiligo in their case reports.^30^ After the publication of article by Pietrzak et al., the association between vitiligo and MetS attracted attention of more researchers.^13^, ^17^ Atas et al. demonstrated increased risk of developing MetS in patients with vitiligo and they found poor clinical features of the disease such as active, extended and segmental vitiligo with an increased duration of time as independent predictors of developing MetS.^13^ Furthermore, Sharma et al. found significant presence of MetS in vitiligo patients. However, they did not demonstrate an association between MetS and severity/activity of vitiligo.^17^

We have demonstrated higher rates of hypertension, hyperlipidemia, obesity, alcohol consumption, vitamin B12 replacement and MetS (for both criteria) in vitiligo patients compared to controls in our study. Additionally, levels of mean FPG, LDL cholesterol, CRP and systolic/diastolic blood pressure were statistically significantly higher in the study group. Furthermore, frequency of MetS increased as the severity/activity of vitiligo increased in our study. The rates of metabolic syndrome were statistically significantly higher in the study group, even after the exclusion of the patients with obesity, diabetes mellitus, Hashimato thyroiditis, vitamin B12 deficiency and alcohol consumption according to both NCEP-ATPIII and IDF criteria. Moreover, mean CRP value in Group C was higher than the mean CRP values in Group A and B which was most probably due to the increased rates of systemic inflammation with the activation of the disease. Our findings are almost consistent with the findings of the studies conducted by Atas et al. and Sharma et al.^13^, ^17^ However, unlike Sharma et al.’s study, we found a significant relationship between vitiligo severity and presence of MetS.^17^ The higher vitamin B12 replacement rate in vitiligo patients was consistent with the findings of the study by Karadag et al.^18^ Furthermore, non-segmental vitiligo might be more associated with chronic inflammation and MetS according to the findings of this study. Similarly, segmental vitiligo was reported to be mostly associated with genetic and autoimmune factors, while MetS was reported to be more frequent in non-segmental vitiligo patients in the literature.^1^, ^3^, ^5^, ^13^ Additionally, higher VASI and VIDA scores in the non-segmental vitiligo patients indicated a more extended and active disease which might be an other reason for the co-existence of chronic inflammation in these patients.^1^, ^3^, ^5^, ^13^

The main strengths of our study were its prospective design, relatively large number of patients included, high number of parameters and homogeneity of laboratory test results. On the other hand, the main limitations of the study were single center experience, absence of more specific oxidative-stress markers, small number of patients in the segmental vitiligo group and lack of long-term follow-up of the patients. Both metabolic syndrome and vitiligo are dynamic syndromes with changing severity over the years and we only tried to demonstrate the cross-sectional association of disease severity with the presence of metabolic syndrome features. Further prospective studies may be carried out to confirm the results of this study.

In conclusion, frequency of MetS was higher in patients with non-segmental vitiligo and the rate was higher in active/severe form of the disease. Thus, controlling risk factors, making life style changes and providing multidisciplinary approach for MetS may be beneficial in patients with vitiligo. Specific therapies for the management of systemic inflammation and oxidative stress should be the targets of physicians who deal with vitiligo.

## Financial support

None declared.

## Authors’ contributions

Efsun Tanacan: Statistic analysis; approval of the final version of the manuscript; conception and planning of the study; elaboration and writing of the manuscript; obtaining, analysis, and interpretation of the data; effective participation in research orientation; intellectual participation in the propaedeutic and/or therapeutic conduct of the studied cases; critical review of the literature; critical review of the manuscript.

Nilgun Atakan Approval of the final version of the manuscript; conception and planning of the study; effective participation in research orientation; critical review of the literature; critical review of the manuscript.

## Conflicts of interest

None declared.
